# Hot-Melt Extrusion as an Advantageous Technology to Obtain Effervescent Drug Products

**DOI:** 10.3390/pharmaceutics12080779

**Published:** 2020-08-17

**Authors:** Ana Luiza Lima, Ludmila A. G. Pinho, Juliano A. Chaker, Livia L. Sa-Barreto, Ricardo Neves Marreto, Tais Gratieri, Guilherme M. Gelfuso, Marcilio Cunha-Filho

**Affiliations:** 1 Laboratory of Food, Drug, and Cosmetics (LTMAC), School of Health Sciences, University of Brasilia, Brasília 70910-900, Brazil; anaaluiza.ln@gmail.com (A.L.L.); ludmila.alvim@gmail.com (L.A.G.P.); tgratieri@gmail.com (T.G.); gmgelfuso@unb.br (G.M.G.); 2 Faculty of Ceilândia, University of Brasília (UnB), Brasília 72220-900, Brazil; chaker@unb.br (J.A.C.); liviabarreto@unb.br (L.L.S.-B.); 3 Laboratory of Nanosystems and Drug Delivery Devices (NanoSYS), School of Pharmacy, Federal University of Goiás, Goiânia 74690-900, Brazil; rnmarreto@gmail.com

**Keywords:** hot-melt extrusion, effervescent drug product, stability, hygroscopicity, disintegration time

## Abstract

Here, we assessed the feasibility of hot-melt extrusion (HME) to obtain effervescent drug products for the first time. For this, a combined mixture design was employed using paracetamol as a model drug. Extrudates were obtained under reduced torque (up to 0.3 Nm) at 100 °C to preserve the stability of the effervescent salts. Formulations showed vigorous and rapid effervescent disintegration (<3 min), adequate flow characteristics, and complete solubilization of paracetamol instantly after the effervescent reaction. Formulations containing PVPVA in the concentration range of 15–20% m/m were demonstrated to be sensitive to accelerated aging conditions, undergoing marked microstructural changes, since the capture of water led to the agglomeration and loss of their functional characteristics. HPMC matrices, in contrast, proved to be resistant to storage conditions in high relative humidity, showing superior performance to controls, including the commercial product. Moreover, the combined mixture design allowed us to identify significant interactions between the polymeric materials and the disintegrating agents, showing the formulation regions in which the responses are kept within the required levels. In conclusion, this study demonstrates that HME can bring important benefits to the elaboration of effervescent drug products, simplifying the production process and obtaining formulations with improved characteristics, such as faster disintegration, higher drug solubilization, and better stability.

## 1. Introduction

Effervescent drug products frequently contain an organic acid source and a hydrogen carbonate salt in their composition, which, in an aqueous medium, react vigorously releasing gas [1,2]. These preparations are hybrids between a solid dosage form, for manufacture and commercialization purposes, and a liquid dosage form, for administration. Moreover, the traditional approach of effervescence is still one of the most efficient and elegant alternatives to facilitate the intake of high drug doses, increase drug release and absorption, and improve taste-masking [1,3,4]. Hence, effervescent medicines account for an important share of the pharmaceutical market involving the delivery of vitamins, analgesics, antacids, anti-inflammatories, and others [5,6].

A notorious weakness of effervescent formulations is their sensitivity to environmental conditions [7]. In particular, the high hygroscopicity of the effervescent components can cause a rapid moisture intake, leading to hydrolysis processes that compromise drug stability. Therefore, strict control of the production area is mandatory, including manufacturing conditions of low temperatures and relative humidity, together with the use of hermetic packaging [8].

Some recent technological advances involving effervescent products have been proposed by means of solvent-consuming and elaborated multistep processes, including freeze-drying, spray-drying, and fluidized bed coating techniques [9,10,11,12,13], which, therefore, are not attractive to the industry.

Hot-melt extrusion (HME), in contrast, is a technology recently incorporated into the pharmaceutical field, which is highly adapted to large-scale production due to its continuous and solvent-free manufacturing [14,15]. The processing of the formulation takes place in a single step by the rotation of screws in controlled conditions of heating and shear [16]. Extruded products have shown excellent flow and tableting characteristics, as well as improved taste, wetting, solubility, and drug stability characteristics [17,18,19,20,21]. Moreover, HME is the perfect match to provide drug filaments for the 3D fused deposition modeling printing of drug products with a modified drug release profile [22].

Thus, we hypothesized here that the HME process could offer advantages for the production of effervescent preparations, possibly by improving both production conditions and product properties. In theory, this process could simplify the production of effervescent products by reducing its steps, as well as obtaining more stable products with better solubility according to the polymeric matrix characteristics. As far as we know, this is the first work involving the development of effervescent drug products by HME.

In this scenario, the purpose of the present study was to assess the feasibility of using HME in the manufacturing of effervescent granules through physicochemical and pharmacopoeial evaluations. Paracetamol was selected as a model drug, considering its extensive use in effervescent commercial products. A combined mixture design was proposed in an approach that allows monitoring the interactions among the components of the formulation, i.e., the polymeric matrices and the disintegrating agents, identifying compositions with optimized results.

## 2. Materials and Methods

### 2.1. Material

Affinisol™ HME 100LV (HPMC, hydroxypropyl methylcellulose, lot ID99015561) was donated by Dow Chemical Company (Midland, MI, USA). Plasdone™ S-630 (PVPVA, poly(vinylpyrrolidone-*co*-vinyl acetate), lot 2095174), and triethyl citrate (TEC, lot 15728) were provided by Ashland Specialty Ingredients (Covington, LA, USA). Parteck^®^ ODT (PTK, lot F1977890) and Ludiflash^®^ (LDF, lot 16800516K0) were granted from Merck (Darmstadt, Germany) and BASF Corp. (Ludwigshafen, Germany), respectively. Paracetamol (lot 1511337) was provided by Purifarma (São Paulo, Brazil). Both citric acid (lot MKCC6083) and sodium bicarbonate (lot SZBE0080V) were afforded by Sigma-Aldrich (St. Louis, MO, USA). All solvents were of analytical grade.

### 2.2. Combined Mixture Design

The effervescent extrudates containing paracetamol were developed according to a combined mixture design planned for using the polymeric matrix (HPMC and PVPVA) and the disintegrating agent (PTK and LDF) as input variables (Table 1) [23,24,25]. The responses were chosen considering important parameters for the production and use of effervescent drug products, i.e., moisture content, disintegration time, compressibility, and drug solubilization. Data were modeled using the Design Expert 11.0 software (Stat-Ease, Minneapolis, MN, USA). The possible models were analyzed by ANOVA one-way, and the fitting model was selected for each response based on *p*-values. The predictive equation for each response was built from stepwise multiple regression analysis [26]. Moreover, a response surface was built for the optimized response considering all responses studied (desirability function from 0 to 1).

### 2.3. Extrusion Conditions and Sample Aging

The HME formulations were comprised of the effervescent salts combined in a stoichiometric proportion (citric acid 24.0%, m/m; and sodium bicarbonate 36.0%, m/m), the plasticizer TEC (5.0%, m/m), the model drug paracetamol (10.0%, m/m), the polymeric matrix HPMC and/or PVPVA (20.0%, m/m), and the disintegrating agent PTK and/or LDF (5.0%, m/m). The last two excipient categories had their proportion in the composition adjusted according to the combined mixture design (Table 1). All components were mixed and then extruded without recirculation in a co-rotating conical twin-screw extruder HME (HAAKE MiniCTW, ThermoScientific, Waltham, MA, USA) at 100 °C in the two heat-points. The parameters of extrusion, such as rotation speed and torque, were selected in order to provide an adequate extrusion flow and material stability (Supplementary Materials Figure S1). The filaments were milled for 20 min at 20,000 rpm using a Hamilton Beach knife mill (Glen Allen, VA, USA) with a stainless-steel blade of 1.6 mm thick. The resulting granules (250–180 µm) were used for the tests.

An accelerated aging treatment was performed in the extrudates placed in Petri glass dishes, which were stored under 75% relative humidity and 40 °C for 24 h [27]. Moreover, each compound used in the formulation, a physical mixture containing paracetamol with citric acid and sodium bicarbonate (PM), and a commercial effervescent drug product containing paracetamol (Sonridor^®^, lot XC3N, GlaxoSmithKline Dungarvan Ltd., Dungarvan, Ireland), underwent the same treatment to serve as controls.

### 2.4. Physicochemical Characterization of the Extrudates

#### 2.4.1. Thermal Analysis

Differential scanning calorimetry (DSC) was performed by a DSC-60 (Shimadzu, Japan) at a heating rate of 20 °C min^−1^ from 25 to 200 °C using 3–5 mg samples placed in aluminum pans. Thermogravimetric analysis (TGA) was performed by a DTG-60H (Shimadzu, Japan) operating at a heating rate of 10 °C min^−1^ from 25 to 500 °C with 3–5 mg samples sited in platinum pans. All analyses were carried out in a nitrogen atmosphere at a flow of 50 mL min^−1^.

#### 2.4.2. Fourier Transform Infrared Spectroscopy (FTIR)

FTIR analyzes were performed in a Varian 640 FTIR spectrometer (Agilent Technologies, Santa Clara, CA, USA). The spectra were recorded between 4000 and 600 cm^−1^ at an optical resolution of 4 cm^−1^ using an attenuated total reflection device. The correlation coefficient between fresh and aged samples [28] was calculated using the FTIR Essential software (Operant LLC, Madison, WIS, USA).

#### 2.4.3. Drug Determination

Quantification of paracetamol in extrudates was performed by a spectrophotometric method using a UV-VIS spectrophotometer UV-1800 (Shimadzu, Japan) operating at 257 nm. The analytical method was validated following the International Conference on Harmonization parameters [29]. Selectivity against polymers and superdisintegrants was appraised, and no statistical interference of the excipients was detected. The linearity correlation coefficient was 0.998, with a slope different from zero and residues randomly distributed without tendency.

#### 2.4.4. Morphological Analysis

The morphological characteristics of the samples were evaluated by optical microscopy using a stereoscope coupled to a video camera (Laborana/SZ—SZT, São Paulo, Brazil) and by a scanning electron microscope (SEM, Jeol, JSM-7001F, Tokyo, Japan) with a previous metallic coating of the samples.

### 2.5. Functional Characterization of the Extrudates

#### 2.5.1. Moisture Content

The moisture content of the samples was assessed in triplicate by gravimetry using a moisture analyzer (MOC63u, Shimadzu, Japan), operating at an equilibrium temperature of 120 °C until reaching a mass variation less than 0.01% m/m for 30 s.

#### 2.5.2. Disintegration Time and Drug Solubilization

Approximately 500 mg of each formulation were placed in 100 mL of water at 25 °C, and the disintegration time was recorded. Samples were considered disintegrated when the granules were disrupted, and the liberation of gas stopped. After 5 min, as established by the pharmacopeia [30], aliquots were collected, filtered, and diluted to determine the drug dissolved using the analytical method previously described. All determinations were performed in triplicate.

#### 2.5.3. Powder Flow Measurement

The compressibility (%) was determined in triplicate using a powder characteristic tester, PT-N (Hosokawa Micron Powder Systems, Summit, NJ, USA), based on the values of aerated density (δa) and packed density (δt) using the following equation [31]:

compressibility (%) = 100 × (δt − δa)/δt,

for samples that could not be analyzed due to their high cohesiveness, a compressibility value equal to 40% obtained for cohesive materials was assigned for model analysis purposes.

### 2.6. Statistical Analysis

The statistical analysis of the data was performed using the GraphPad Prism 8 (San Diego, CA, USA). Results were analyzed using two-way ANOVA followed by Tukey post-test, since the data passed the normality test, showing parametric behavior. The level of significance (*p*) was fixed at 0.05.

## 3. Results and Discussion

### 3.1. Initial Trials and Extrusion Setup

Several hydrophilic thermoplastic polymers commonly used in HME combined with effervescent components were tested in the usual extrusion conditions of each material. The first challenge was to achieve a satisfactory extrusion consuming a minimum amount of polymer since the salts that trigger the gas release need to be in high proportion in effervescent products [32]. For some polymers, this was achieved using only 20% m/m of these components.

Initial trials demonstrated that extrusion temperatures above 100 °C promoted the decomposition of sodium bicarbonate, leading to gas release followed by the expansion and darkening of the filament. Although the thermal decomposition of this salt occurs at 120 °C [33], the extrusion shear anticipated this event to lower temperatures. Therefore, a plasticizer was added (5% m/m) to reduce the extrusion temperature to 100 °C. TEC was selected for being a versatile plasticizer agent for several HME polymers [34].

The next step was to ensure that the extruded granules had the disintegration time within 5 min, the time required for an effervescent drug product [30]. Most of the polymeric matrices did not produce granules with the required performance, resulting in dense materials that prevented the granule from triggering the effervescent reaction.

The addition of co-processed excipients used in orodispersible tablets was evaluated as an alternative. In particular, two products that combined superdisintegrants with mannitol, a sugar alcohol with high aqueous solubility, were used (PTK and LDF). This combination of components proved to be very effective in accelerating the disintegration of the effervescent granules with different polymeric matrices and, therefore, were introduced as a variable in the experimental design. In fact, the combined mechanisms of solubilization and swelling of the granule achieved by using these materials led to a more intense effervescent reaction that rapidly disintegrates the extrudates. Moreover, other advantages of using these materials for an effervescent product could be listed, such as improving taste-masking, flowability, and tableting, as well as reducing the solid residual after disintegration [35].

For further formulation development, two polymeric matrices were selected with similar glass transition temperature, allowing us to combine both in the proposed mixture design: PVPVA and HPMC. These polymers show glass transition at 109 and 115 °C, respectively [36,37].

The trials revealed components with promising potential for obtaining, for the first time, an effervescent drug product by HME; however, other technological challenges still needed to be addressed. Besides fulfilling the disintegration requirements, the HME matrix must gather other attributes such as chemical and physical stability, adequate rheology, and not hindering the drug solubilization.

Accordingly, a combined mixture design involving the two components that have the most substantial influence on these responses—the polymeric matrix and the disintegrating agent—was applied (Table 1) to understand how the formulation composition could impact its physicochemical properties and, after that, the pharmaceutical performance.

The use of TEC, in addition to the plasticizing effects of citric acid and the drug itself, allowed the extrusion of all formulations at 100 °C. The screws rotation was selected in order to minimize the risk of effervescent salts degradation caused by shear, as well as promoting the homogeneous mixture of the materials; thus, moderate screw rotation speeds were used (50–75 rpm), adjusted to a maximum torque of 0.3 Nm (Table 1). Otherwise, higher torque triggered the decomposition of the effervescent components with the expansion of the extruded filament due to gas release during the extrusion. For the formulations containing HPMC, a low rotation of the screws (50 rpm) had to be used to increase the material extrusion time and, consequently, produce a homogeneous filament. In contrast, PVPVA formulations were processed at 75 rpm to produce a filament with appropriate viscoelastic characteristics for granulation.

Therefore, the most favorable extrusion conditions were obtained using PVPVA, which allowed the extrusion using lower torque, producing more flexible and porous filaments. On the other hand, formulations using only HPMC as a polymeric matrix (F5, F7, and F11) exhibited denser and more compact filaments (Supplementary Materials Figure S1).

### 3.2. Physicochemical Evaluation of the Extrudates

In the DSC results of the physical mixtures, despite the dilution of paracetamol in the formulations and the interference of the large decomposition endotherm of sodium bicarbonate, a peak of around 170 °C corresponding to the drug melting was assigned under the performed analysis conditions. Moreover, the enthalpy involved in this event was significantly reduced due to the partial solubilization of the drug in the polymeric matrix during the analysis itself (Table 2). In contrast, the fresh HME samples did not exhibit the paracetamol melting peak, suggesting the amorphization of the drug due to the HME process (Supplementary Materials Figure S2). Similarly, the aged extrudates showed no drug melting event, indicating the absence of drug recrystallization after aging. Similar results were reported in the literature using paracetamol; however, upon extrusion temperatures above 120 °C [38].

The polymeric matrices and disintegrating agents alone exhibited high thermal stability according to the TGA results, with an initial decomposition temperature above 230 °C. Additionally, paracetamol showed a decomposition temperature above 195 °C. In contrast, the effervescent components are thermosensitive, i.e., citric acid decomposes just after its melting at around 170 °C, while sodium bicarbonate decomposes from 120 °C. Thus, preserving the stability of effervescent salts during the HME process was the major challenge to overcome.

All extrudates obtained in the experimental design showed initial decomposition temperatures in the range of 120–130 °C (Table 2), with a mass loss profile in several stages corresponding to the sum of the excipients and drug decomposition profile (around 60–70% of the sample until 500 °C). These results indicate chemical compatibility between the components of the formulation and suggest the preservation of the material stability after the extrusion process [39].

**Figure 1:**
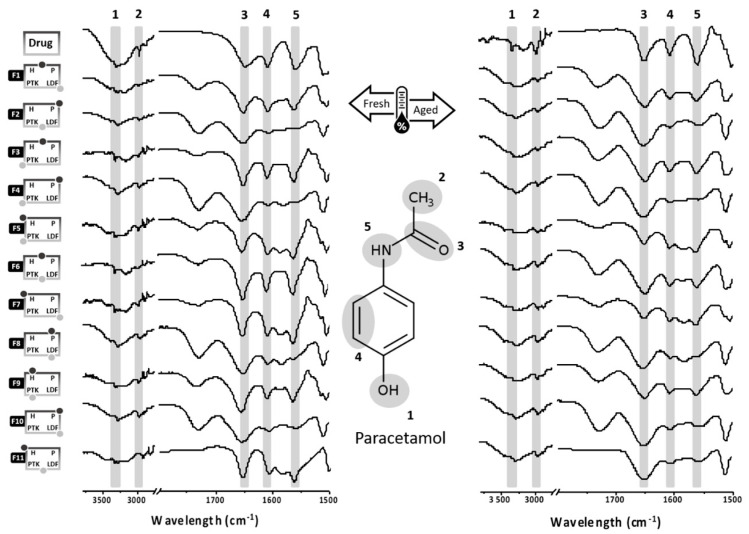
FTIR of the paracetamol as supplied and the effervescent extrudates obtained according to a combined mixture design before and after aging.

The paracetamol functional groups could be identified from the FTIR spectra of the fresh extrudates, corroborating the stability of the formulations after extrusion (Figure 1). Moreover, the drug content of all formulations fulfills the pharmacopoeial range of 90–110% [40]. Moreover, a high correlation index was observed among the spectra of the fresh and aged samples for all extrudates (*r* > 0.9, Table 2), providing evidence that the chemical integrity of these systems would also be maintained during the shelf period [28].

**Figure 2:**
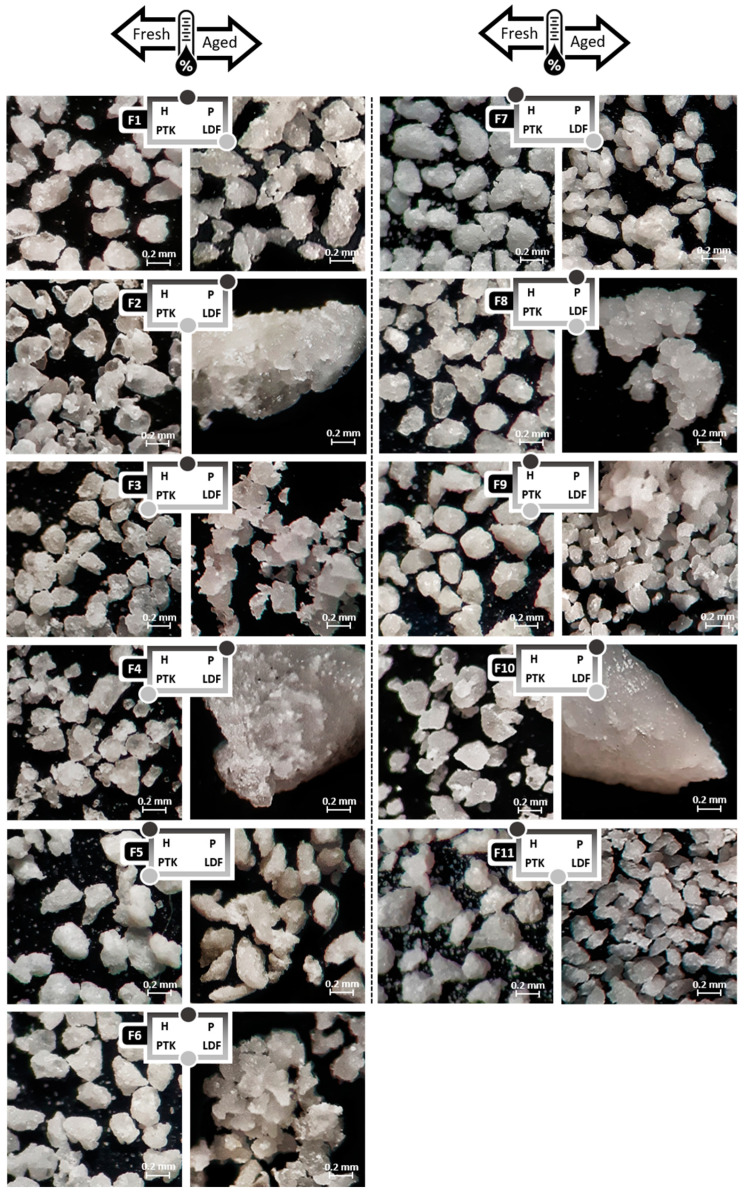
Optical microscopy photomicrographs of effervescent extrudates obtained according to a combined mixture design before and after aging at a 45× magnification.

**Figure 3:**
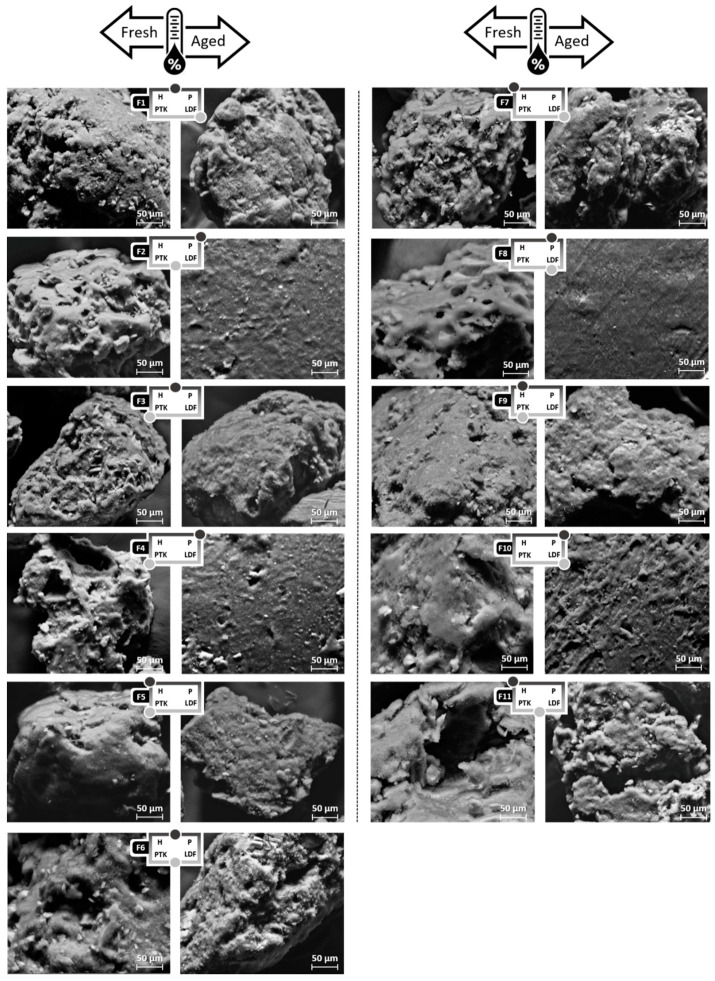
SEM photomicrographs of effervescent extrudates obtained according to a combined mixture design before and after aging at a 1000× magnification.

Regarding the morphological aspects, the extrudate granules showed a regular size and a rounded shape (Figure 2). SEM photomicrographs of the surface of the granules showed that PVPVA-based extrudates are more porous, while those obtained using HPMC presented a more regular and compact surface (Figure 3). The thermal aging associated with a high relative humidity exposure caused intense microstructural changes in some formulations, according to the polymeric matrix used. In particular, extrudates containing high proportions of PVPVA underwent particle agglomeration caused by a plasticizer effect exerted by the moisture captured by these samples. In fact, SEM photomicrographs corroborate this hypothesis showing that in these samples (F2, F4, F8, and F10), the polymeric matrix formed a film that extended almost continuously on the extrudate.

### 3.3. Pharmaceutical Performance of the Extrudates

The statistical evaluation of the combined mixture design for the selected responses led to significant predictive models (*p* < 0.0001) and high correlation coefficients (*r*^2^ > 0.8). Moreover, the adequate precision parameter, which determines the range of a predicted response relative to its associated error, exhibited values higher than 10, allowing navigation in space design [41].

**Figure 4:**
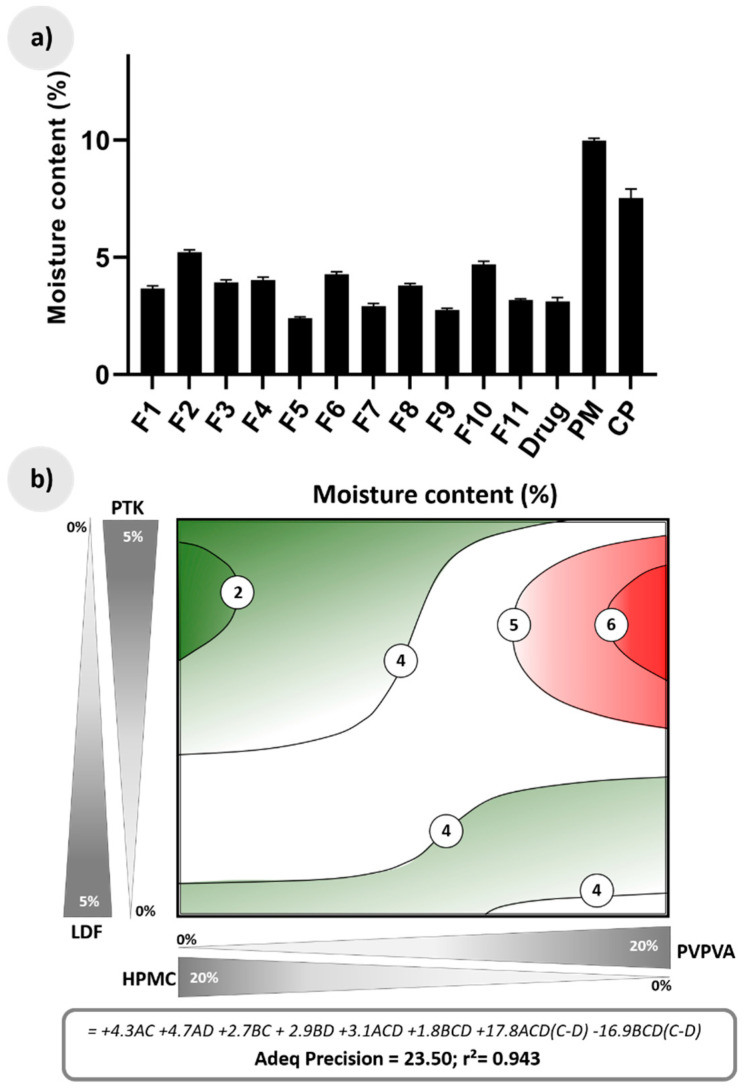
(**a**) Moisture content (%) of the drug as supplied, the physical mixture containing drug and effervescent salts (PM), commercial drug product (CP), and effervescent extrudates after aging; (**b**) Response surface for moisture content according to a linear-cubic model, together with its predictive equation (A = PVPVA, B = HPMC, C = PTK, and D = LDF). The green regions show compositions with low moisture levels, and the red regions indicate compositions with high moisture levels.

The determination of water content in the HME formulations, as well as in the selected controls after aging is shown in Figure 4a. The uptake of water by the commercial effervescent product containing paracetamol was about 7.5%, while the physical mixture of the drug with the effervescent salts (PM) showed an even higher value (10.0%). In contrast, HME extrudates exhibited low moisture levels between 2.5–5.0%.

In fact, the well-known hygroscopicity of effervescent components is one of the main obstacles faced in the production of effervescent drug products, demanding productive areas with strict environmental humidity controls [11,42]. In this sense, the higher resistance of HME extrudates to water uptake is an important industrial advantage of these products. Additionally, the previous dry treatment of the raw material to be used in the manufacturing of the effervescent drug product would not be necessary using this technology, as the residual moisture of the raw materials can eventually assist HME processing due to water plasticizer effect, without bringing negative consequences to the extrudates, once this residual water ends up being evaporated at the end of the extrusion. In fact, the moisture of fresh extrudates was within the range of 0.5–2.0%.

The moisture levels of the HME granules after aging was linked to the formulation composition, being mainly influenced by the polymeric matrix, as illustrated on the response surface of Figure 4b. The regions in which the formulation has higher proportions of PVPVA lead to granules with higher water content (regions in red in Figure 4b). Furthermore, the coefficients of the terms containing this polymer have values almost twice as high as those involving the HPMC. Previous studies have shown that PVPVA can be a moisture-sensitive ingredient, especially under conditions of high relative humidity [43]. The response surface also shows compositions of the extrudate in which the moisture levels are quite low (around 2%), containing mostly HPMC and PTK (green regions in Figure 4b).

**Figure 5:**
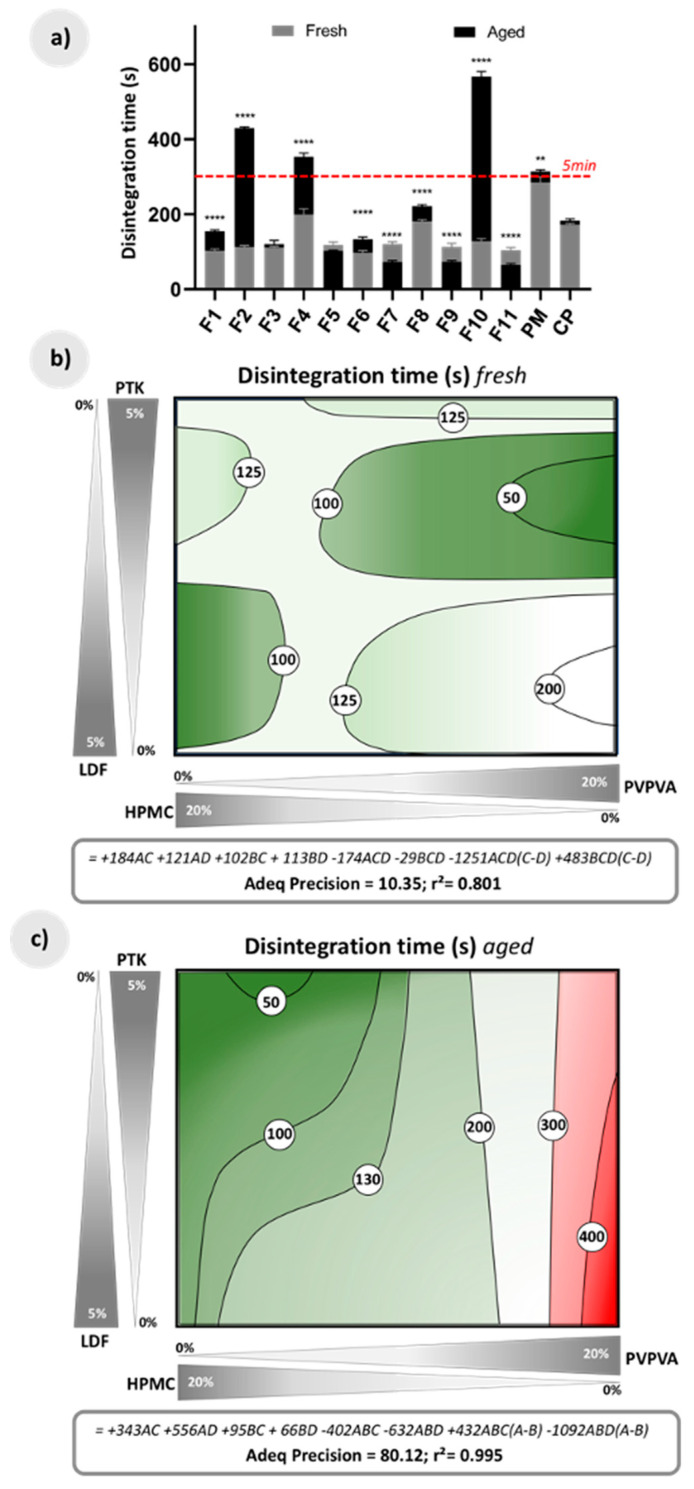
(**a**) Disintegration time (s) of fresh and aged physical mixture of drug and effervescent salts (PM), commercial drug product (CP), and effervescent extrudates; (**b**) Response surface for disintegration time in fresh extrudates according to a linear-cubic model, together with its predictive equation; (**c**) Response surface for disintegration time in aged extrudates according to a cubic-linear model, together with its predictive equation. A = PVPVA, B = HPMC, C = PTK, and D = LDF. The red dotted line in (**a**) marks the pharmacopoeial limit; the green regions in (**b** and **c**) show compositions within the sanitary limit, and the red regions in (**c**) indicate compositions outside this limit. ^****^ (*p* < 0.0001), ^**^ (*p* < 0.005).

As previously mentioned, a mandatory condition for effervescent medicines is to rapidly disintegrate in the water at room temperature, specifically within 300 s [30]. During the extrudates development, a densification of the polymeric matrix promoted by HME was observed, which could have hindered the reaction of the effervescent components. Even so, in the experimental design presented here, all the compositions met this requirement satisfactorily (grey bars of Figure 5a). Furthermore, most of the compositions showed faster disintegration than the commercial effervescent product (CP) and the physical mixture (PM), in which the effervescent salts are free to trigger the effervescent reaction in the aqueous medium. These results demonstrate the promising potential of HME in developing effervescent products conditioned by the use of suitable experimental conditions.

The response surface for this parameter in the freshly prepared HME granules shows that the polymer matrix PVPVA is very sensitive to the disintegrating agent used, with a disintegration time of less than 50 s when PTK is used, and higher than 200 s when using LDF (Figure 5b). The disintegration time of extrudates containing mostly HPMC, in turn, is practically unaffected by the disintegrating agent, resulting in granules with disintegration in around 100 s.

When forced aging conditions challenged the extrudates, formulations containing more than 15% of PVPVA had a noticeable reduction in the intensity of the effervescent reaction and increased disintegration times, failing the sanitary limits for this response (regions in red in Figure 5c). The microstructural changes that occurred with these samples led to non-porous granules, as discussed in Section 3.2, and ultimately produce a negative impact on their disintegration capacity. The polymeric matrix interacts with components of the formulation, making them inaccessible to water, and even annulling the effect of disintegrating agents.

In contrast, the presence of the HPMC polymer in at least 5% seems to preserve the disintegration properties of the granules. In fact, in the predictive equation, the positive value coefficients involving this polymer are up to five times lower than in the terms involving PVPVA (Figure 5c).

**Figure 6:**
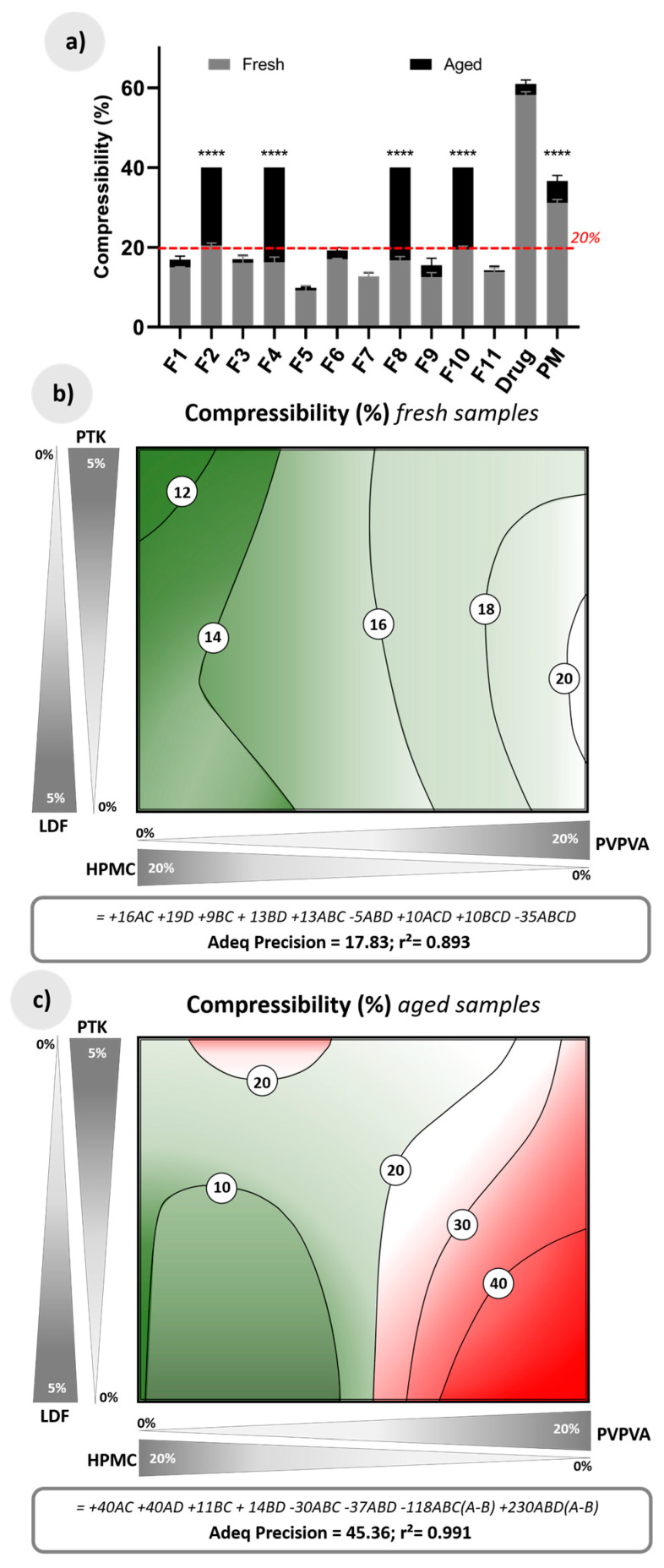
(**a**) Compressibility (%) of fresh and aged raw material drug, physical mixture of drug and effervescent salts (PM), and effervescent extrudates; (**b**) Response surface for compressibility in fresh extrudates according to a quadratic-quadratic model, together with its predictive equation; (**c**) Response surface for compressibility in aged extrudates according to a cubic-linear model, together with its predictive equation. A = PVPVA, B = HPMC, C = PTK, and D = LDF. The red dotted line in (**a**) marks the limit for an appropriate flow; the green regions in (**b**) and (**c**) show compositions within particle flee flow, and the red regions in (**c**) indicate compositions with cohesive particles. ^****^ (*p* < 0.0001).

All fresh extrudates showed adequate rheological characteristics for pharmaceutical production (gray bars of Figure 6a), with compressibility values below 20% [44] and considerable improvement of the deficient flow characteristics of paracetamol as supplied. In fact, the HME process promotes the densification of the solid material, and the plastic polymer matrix favors the cut in the granulation process originating free-flow particles [45,46].

The flow results are strongly influenced by the composition of the granulate (Figure 6b). The excellent flow was achieved when using mostly HPMC and the disintegrant PTK (compressibility <12%), while good or fair flow was obtained using PVPVA in any composition of the disintegrating agents studied (compressibility 16–20%).

The structural changes caused by the aging of samples containing PVPVA in proportions higher than 10%, which initiated the aggregation of the granules (Figure 2), made these granules extremely cohesive, confirming the loss of their functionality. Figure 6c shows the regions in red, which represent compositions more sensitive to rheological problems due to water intake (coefficients of the predictive equation involving PVPVA are up to four times greater than those that include HPMC).

**Figure 7:**
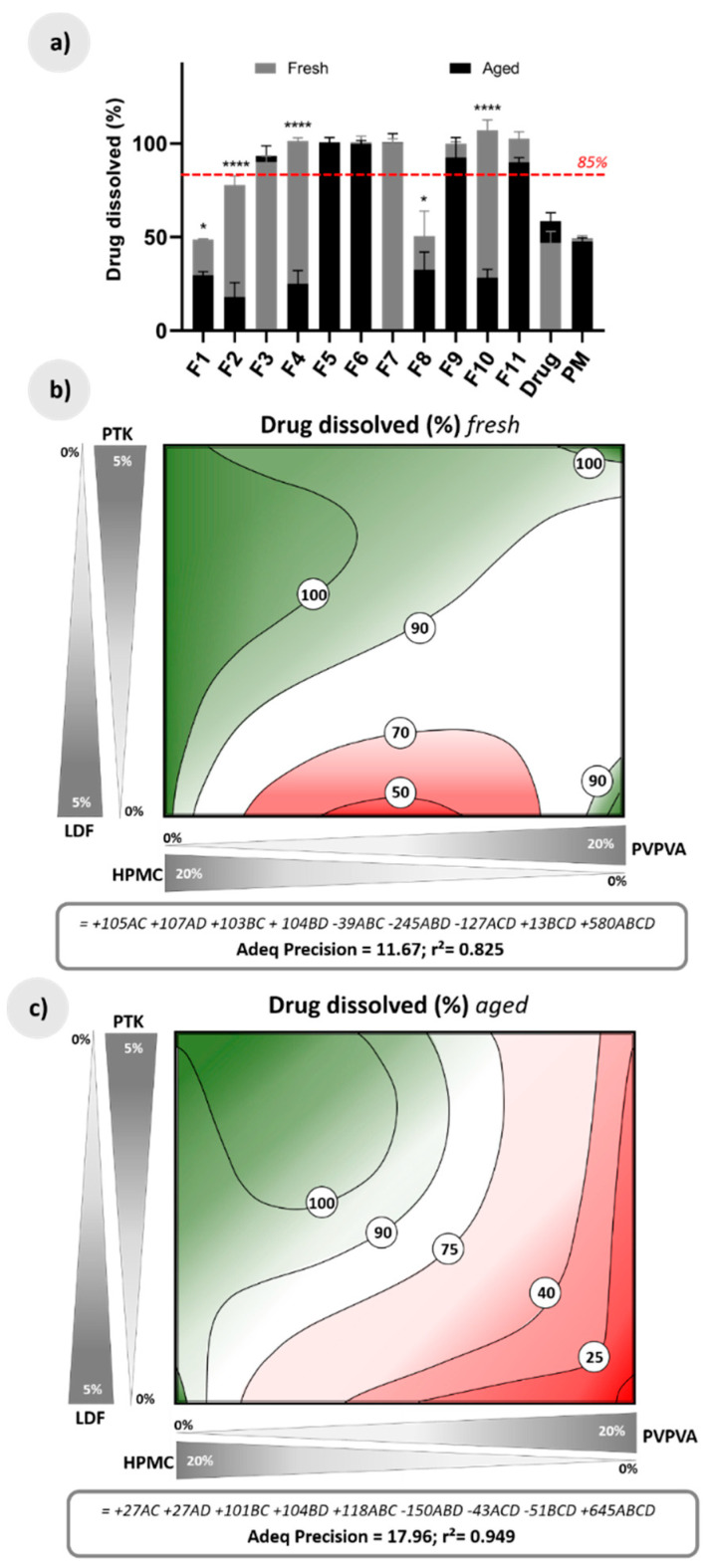
(**a**) Drug dissolved (%) of fresh and aged raw material drug, physical mixture of drug and effervescent salts (PM), and effervescent extrudates; (**b**) Response surface for drug dissolved in fresh extrudates according to a quadratic-quadratic model, together with its predictive equation; (**c**) Response surface for drug dissolved in aged extrudates according to a quadratic-quadratic model, together with its predictive equation. A = PVPVA, B = HPMC, C = PTK, and D = LDF. The red dotted line in (**a**) marks the limit for drug solubilization according to the FDA; the green regions in (**b**) and (**c**) show compositions with appropriate solubilization, and the red regions in (**c**) indicate compositions with inappropriate solubilization. ^****^ (*p* < 0.0001), ^*^ (*p* < 0.05).

An important aspect in an effervescent preparation is the drug solubilization in the liquid medium after effervescence, ensuring an adequate bioavailability. Paracetamol, chosen as a model drug, has high aqueous solubility (14.2 mg/mL [47]), even so, after 5 min, only 50% of the dose was dissolved in water at 25 °C from the raw material (Figure 7a), probably due to the slow dissolution of its crystals and the low wetting of the solid particles. Hence, simply mixing the effervescent components with the drug, despite the effervescence reaction, does not improve this result.

In contrast, the HME processing promoted drug amorphization, which, combined with a hydrophilic polymeric base and the effervescent reaction, led to the complete solubilization of paracetamol in a short time for most of the extrudates (Figure 7a). For some extruded compositions, however, the solubilization of the drug was similar to that of the drug as supplied (about 50%). In the response surface for fresh samples, this happened specifically in mixtures containing the two polymers in equal proportion and using LDF (red regions in Figure 7b). In fact, the term involving both polymers and PTK (ABD) in the predictive equation was negative and had a high coefficient (Figure 7b). Despite the rapid disintegration of these systems (Figure 5b), the divided concentration of each polymeric matrix in this region of the design is not sufficient to promote a marked interaction of the drug with any of the polymers, annulling its action in assisting the solubilization of paracetamol.

After forced aging of the samples, some extrudates lost the ability to dissolve the drug rapidly (F1, F4, and F10, dark bars in Figure 7a). As a comparison, the FDA recommends that immediate-release solid oral dosage forms should dissolve at least 85% of their dose at the end of the assay [48]. These extrudates, which contain higher proportions of PVPVA, underwent major structural changes as previously discussed, hindered matrix disintegration, and, consequently, the drug solubilization. The response surface shows that the combination of PVPVA and LDF intensifies this effect, resulting in less than 20% of paracetamol dissolved before the patient intake (red regions in Figure 7c).

Still, formulations containing mostly HPMC preserve their high solubilization capacity after aging, regardless of the disintegrating agent used. In fact, this polymer showed in the predictive equation coefficients values up to four times higher than the coefficients related to PVPVA (Figure 7c).

**Figure 8:**
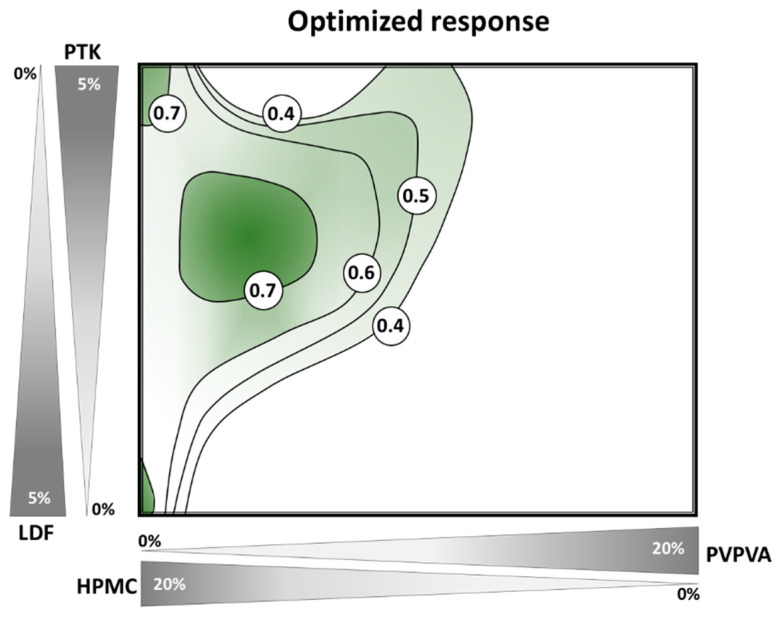
Response surface for the optimized response (desirability index) of effervescent formulations produced by HME, considering moisture content, disintegration time, compressibility, and drug solubilization. The green regions show compositions within better pharmaceutical performance.

Finally, all the responses were integrated in order to produce an optimized response surface with outcomes following the pharmacopoeial parameters. Hence, formulations containing only HPMC, as a polymeric matrix, with any of the disintegrating agents, as well as mixtures of both polymers HPMC/PVPVA and PTK led to suitable results (green regions in Figure 8). Importantly, this wide range of possible formulation compositions facilitates the scale-up production, allowing adjustments in the formulation in large-scale production. In addition, the HME process usually presents a more simplified scale-up than other industrial processes [49]. Particularly, HME production of effervescent products could be associated to an open zone in the barrel, therefore any water vapors could be eliminated by the degassing process of the material, producing filaments with even lower moisture content [50,51].

## 4. Conclusions

This study demonstrates that the HME process can bring great benefits to the production of effervescent drug products, simplifying the production process, involving fewer manufacturing steps, and originating intermediate products with more favorable flow characteristics for a tablet transformation. Furthermore, the final product may present better characteristics than commercial effervescent drug products prepared by traditional processes, such as faster disintegration, higher drug solubilization, and better stability in conditions of high relative humidity storage.

Nevertheless, the extrusion conditions, as well as the qualitative and quantitative composition, play a crucial role in the viability of this technology. In this sense, the use of quality-by-design tools as experimental mixture designs can accurately identify the concentration ranges and combinations of excipients that can lead to optimal pharmaceutical product performance.

## Figures and Tables

**Table 1:** Summary of the formulation composition according to the combined mixture design and their extrusion conditions. In formulations representation, H refers to HPMC and P to PVPVA.

Formulation Representation	Formulation Components (%, m/m)				Extrusion Conditions	
	PVPVA	HPMC	PTK	LDF	Rotation (rpm)	Torque (*Nm*)
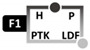	10.0	10.0	0.0	5.0	50	0.20
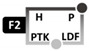	20.0	0.0	2.5	2.5	75	0.15
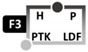	10.0	10.0	5.0	0.0	50	0.20
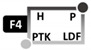	20.0	0.0	5.0	0.0	75	0.15
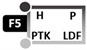	0.0	20.0	5.0	0.0	50	0.30
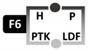	10.0	10.0	2.5	2.05	50	0.20
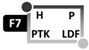	0.0	20.0	0.0	5.0	50	0.30
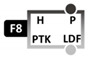	15.0	5.0	1.25	3.75	50	0.20
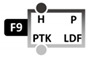	5.0	15.0	3.75	1.25	50	0.20
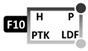	20.0	0.0	0.0	5.0	75	0.15
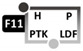	0.0	20.0	2.5	2.5	50	0.30

**Table 2:** Thermogravimetric analysis (TGA) of the extrudates including the sample initial decomposition temperature (°C), the mass loss (%), and the number of decomposition phases occurring up to 500 °C, together with the differential scanning calorimetry (DSC) results for Tpeak (°C) and heat (J/g) of paracetamol melting in physical mixtures (PM) and the FTIR correlation coefficient between fresh and aged extrudates.

Formulation Representation	TGA			DSC		FTIR
	Initial Decomposition Temperature (°C)	Mass Loss (%)	Number of Decomposition Steps	Tpeak (°C)	Heat (J/g)	Correlation Coefficient
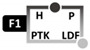	126.1	62.4	6	167.9	−1.44	0.9715
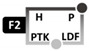	124.4	71.5	6	167.8	−1.07	0.9558
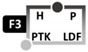	125.2	65.1	6	171.5	−1.47	0.9112
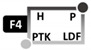	125.8	67.5	5	170.3	−1.42	0.9803
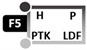	134.2	61.5	4	170.8	−0.23	0.9471
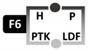	126.5	63.2	6	165.2	−1.19	0.9032
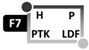	131.3	64.0	5	171.4	−3.51	0.9349
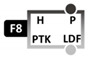	127.5	77.1	5	166.5	−2.36	0.9741
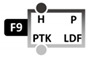	126.2	60.6	5	167.6	−1.91	0.8668
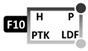	123.8	68.6	5	165.4	−0.28	0.9946
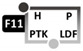	132.2	59.5	4	165.7	−0.21	0.9556

## Supplementary Materials

The following are available online at https://www.mdpi.com/1999-4923/12/8/779/s1, Figure S1: Optical microscopy photomicrographs of the longitudinal and transversal view of the filaments at a 30× magnification, Figure S2: DSC curve of paracetamol as supplied and the fresh extrudates. Dark blue dotted lines show citric acid degradation and light blue dotted lines show sodium bicarbonate degradation.
