# Supplementary material for: Hot-Melt Extrusion as an Advantageous Technology to Obtain Effervescent Drug Products
Source: Pharmaceutics. 2020 Aug 17;12(8):779. doi: 10.3390/pharmaceutics12080779 (PMC7464369; doi:10.3390/pharmaceutics12080779)
Supplement: Supplementary file 1 [file pharmaceutics-12-00779-s001.pdf]

pharmaceutics

MDPI

Article

Supplementary Materials: Hot-Melt Extrusion as an Advantageous Technology to Obtain Effervescent Drug Products

Ana Luiza Lima, Ludmila A. G. Pinho, Juliano A. Chaker, Livia L. Sa-Barreto, Ricardo Neves Marreto, Tais Gratieri, Guilherme M. Gelfuso and Marcilio Cunha-Filho

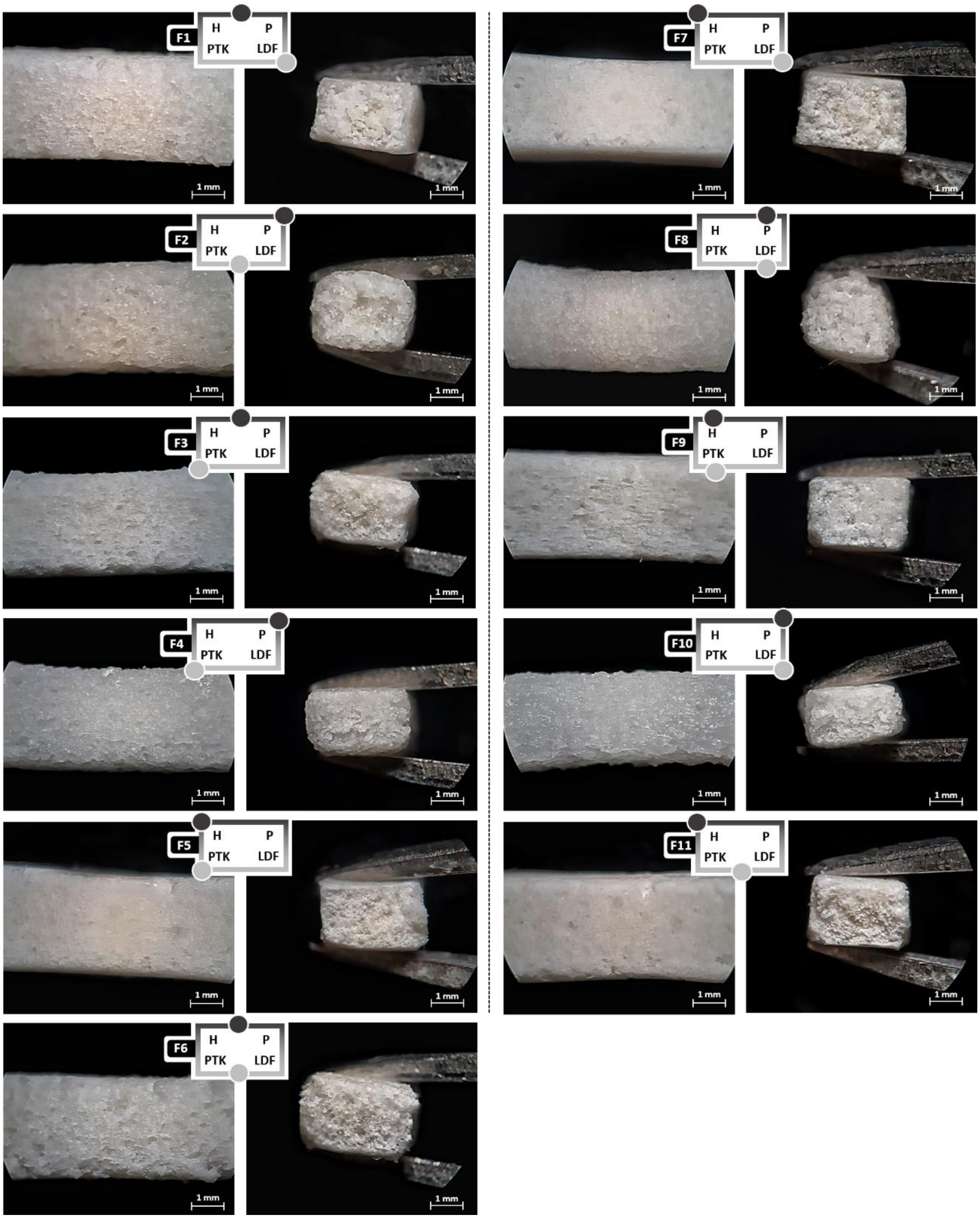

Figure S1. Optical microscopy photomicrographs of the longitudinal and transversal view of the filaments at a 30× magnification. .

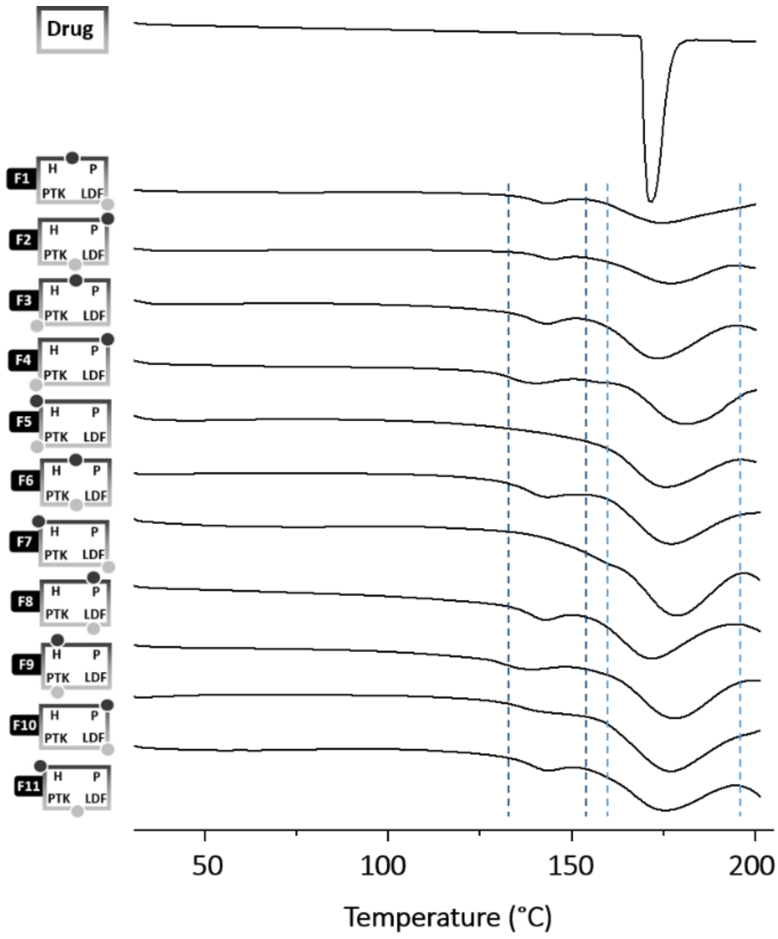

Figure S2. DSC curve of paracetamol as supplied and the fresh extrudates. Dark blue dotted lines show citric acid degradation and light blue dotted lines show sodium bicarbonate degradation.
